# Supplementary material for: High-frequency FeSiAl-based soft magnetic composites via simultaneously suppressed eddy and hysteresis losses
Source: Nat Commun. 2025 Oct 29;16:9563. doi: 10.1038/s41467-025-64794-0 (PMC12572122; doi:10.1038/s41467-025-64794-0)
Supplement: Supplementary file 1 — Supplementary Information [file 41467_2025_64794_MOESM1_ESM.pdf]

Supplementary Information

High-frequency FeSiAl-based soft magnetic composites via simultaneously suppressed eddy and hysteresis losses

Hongxia Li[1,#], Yixing Li[2,#], Rongzhi Zhao[1,*], Sateesh Bandaru[1], Zhenhua Zhang[1], Hong Pan[1], Jintao Lin[1], Zhaoyuan Liu[1], Xiaolian Liu[1], Chenglong Hu[1], Pengfei Guan[1], Anjian Pan[1], Erpan Zhang[1], Zhong Li[1], Huawei Rong[1], Xuefeng Zhang [1,*]

[1]Institute of Advanced Magnetic Materials, College of Materials and Environmental Engineering, Hangzhou Dianzi University, Hangzhou 310018, China

[2] Key Laboratory for Anisotropy and Texture of Materials (MOE), School of Materials Science and Engineering, Northeastern University, Shenyang 110819, China.

[#] These authors contributed equally.

* Corresponding author E-mail: zhaorz@hdu.edu.cn, zhang@hdu.edu.cn

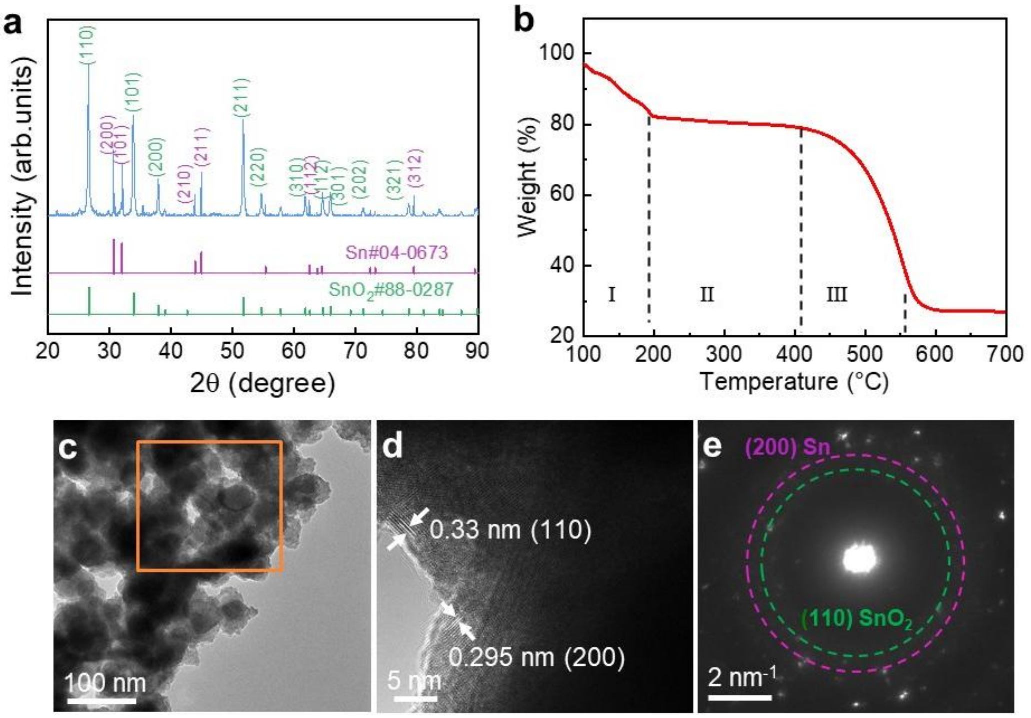

Supplementary Fig. 1. Formation of SnO_2 and Sn. a, XRD patterns of $SnCl_2$ solution after annealing, confirming the formation of SnO_2 and Sn. **b,** TGA curve of $SnCl_2 \cdot 2H_2O$ powder, where $SnCl_2 \cdot 2H_2O$ is transformed to $Sn(OH)_2$ in step I, $Sn(OH)_2$ is transformed to SnO in step II, and SnO is transformed to SnO_2 and Sn in step III, indicating the disproportionation reaction of SnO. **c, d,** TEM and HRTEM image of the $SnCl_2$ solution after annealing, where lattice fringes of 0.33 nm and 0.295 nm correspond to the (110) plane of SnO_2 and (200) plane of Sn, respectively. **e,** SAED pattern of the orange box in c, confirming the formation of SnO_2 and Sn. Source data are provided as a Source Data file.

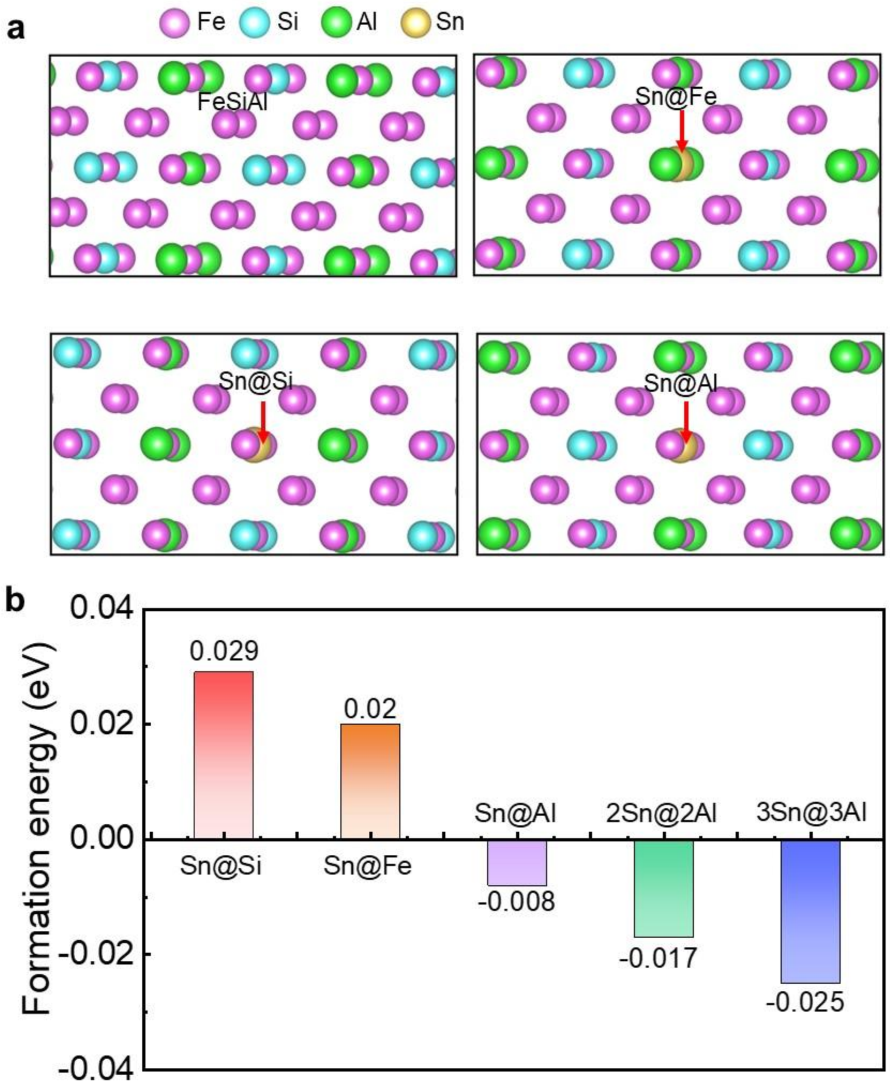

Supplementary Fig. 2. Formation energy simulation. a, Schematic models of FeSiAl, Sn@Fe, Sn@Si, and Sn@Al. **b,** Formation energy of Sn@Si, Sn@Fe, Sn@Al, 2Sn@2Al and 3Sn@3Al models. Source data are provided as a Source Data file. The formation energy (E_{form}) of different models is calculated by the following equations, respectively:

$$E_{form1} = E_{[Sn_1Fe_{95}Si_{16}Al_{16}]} - E_{[Fe_{96}Si_{16}Al_{16}]} + (\mu_{Fe} - \mu_{Sn}) \quad (1)$$

$$E_{form2} = E_{[Sn_1Fe_{96}Si_{15}Al_{16}]} - E_{[Fe_{96}Si_{16}Al_{16}]} + (\mu_{Si} - \mu_{Sn}) \quad (2)$$

$$E_{form3} = E_{[Sn_xFe_{96}Si_{16}Al_{16-x}]} - E_{[Fe_{96}Si_{16}Al_{16}]} + x(\mu_{Al} - \mu_{Sn}) \quad (3)$$

Where $E_{[Fe_{96}Si_{16}Al_{16}]}$ is energy of the FeSiAl super cell, $E_{[Sn_xFe_{96}Si_{16}Al_{16-x}]}$ represents the energies of different Sn@Al substitutions (x=1 to 3), μ_{Fe}, μ_{Si}, μ_{Al} and μ_{Sn} are the chemical potentials of Fe, Si, Al and Sn, respectively.

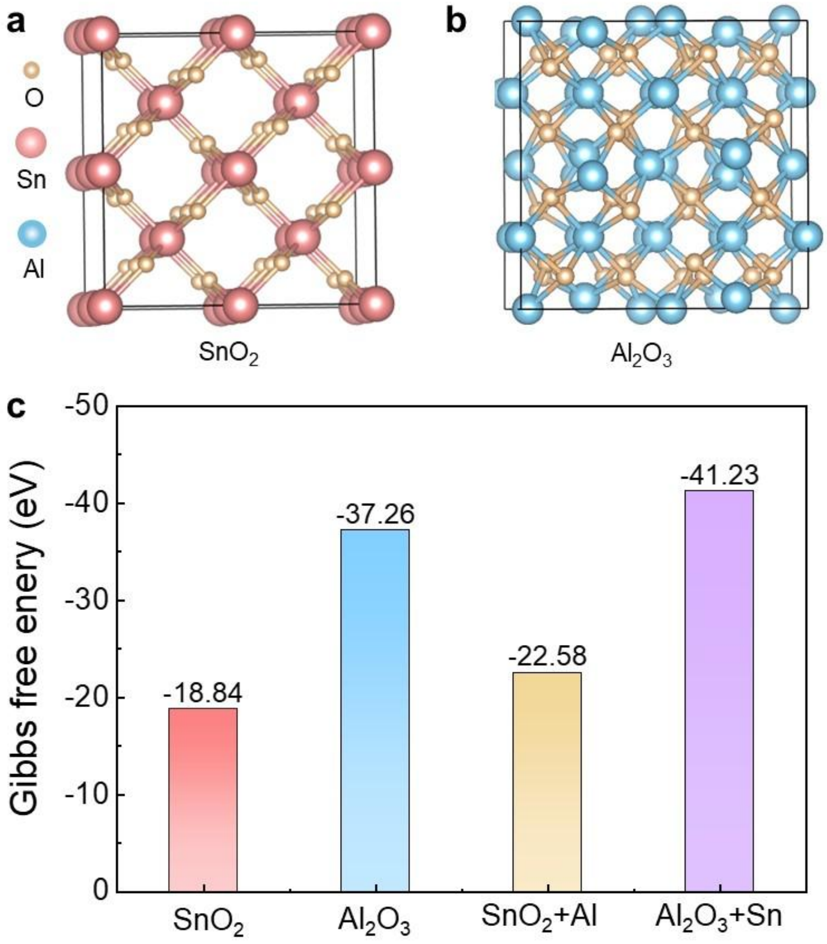

Supplementary Fig. 3. Gibbs free energy calculation. a, b, The simulation model of the (a) tetragonal SnO_2 and (b) cubic Al_2O_3. **c,** Gibbs free energy of SnO_2, Al_2O_3, SnO_2+Al and Al_2O_3+Sn. Source data are provided as a Source Data file. To investigate the feasibility of formation of Al_2O_3+Sn from SnO_2+Al, the formation energy (E_{form}) is calculated by the following equation:

$$E_{\mathrm{form}} = E_{[\mathrm{Al_2O_3}]} + x\mu_{\mathrm{Sn}} - [E_{[\mathrm{SnO_2}]} + x\mu_{\mathrm{Al}}] \quad (4)$$

Where $E_{[\mathrm{Al_2O_3}]}$ represents the unit cell energy of cubic Al_2O_3 system, $E_{[\mathrm{SnO_2}]}$ is the unit cell energy of cubic SnO_2, $\mu_{Sn}$and μ_{Al} are the chemical potentials of Sn and Al. These energies are computed from the corresponding stable states.

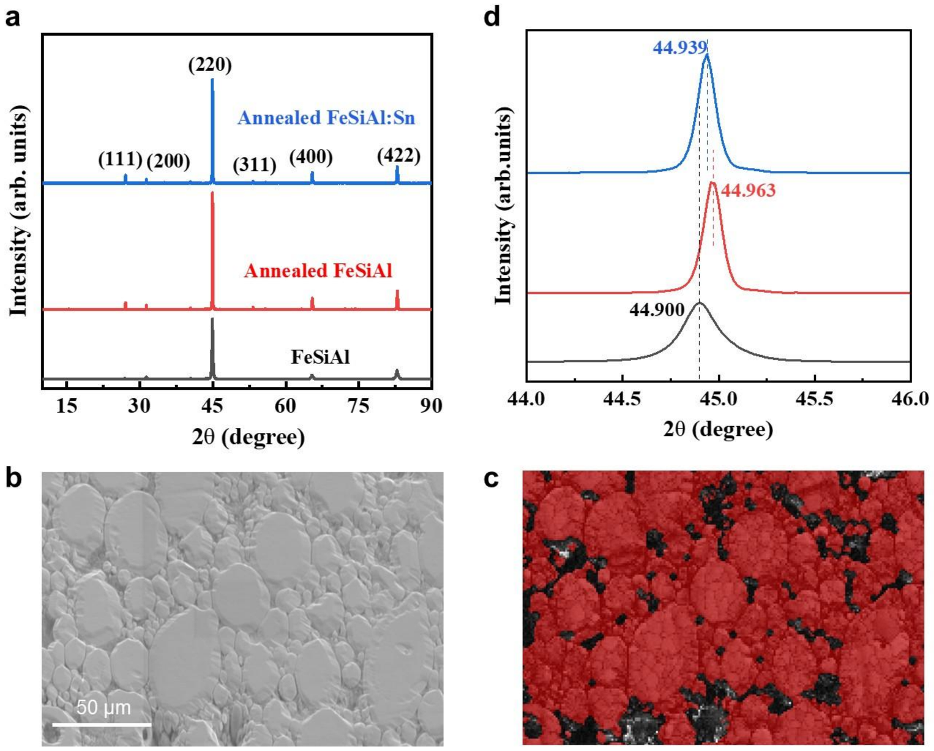

Supplementary Fig. 4. FeSiAl:Sn with DO3 structure. a, XRD patterns of different FeSiAl and FeSiAl:Sn particles. **b,** SEM cross sectional image of FeSiAl:Sn-0.8 SMC. **c,** The corresponding EBSD phase map of FeSiAl:Sn-0.8 SMC with single FCC phase. **d,** (220) diffraction peaks of different particles. Source data are provided as a Source Data file.

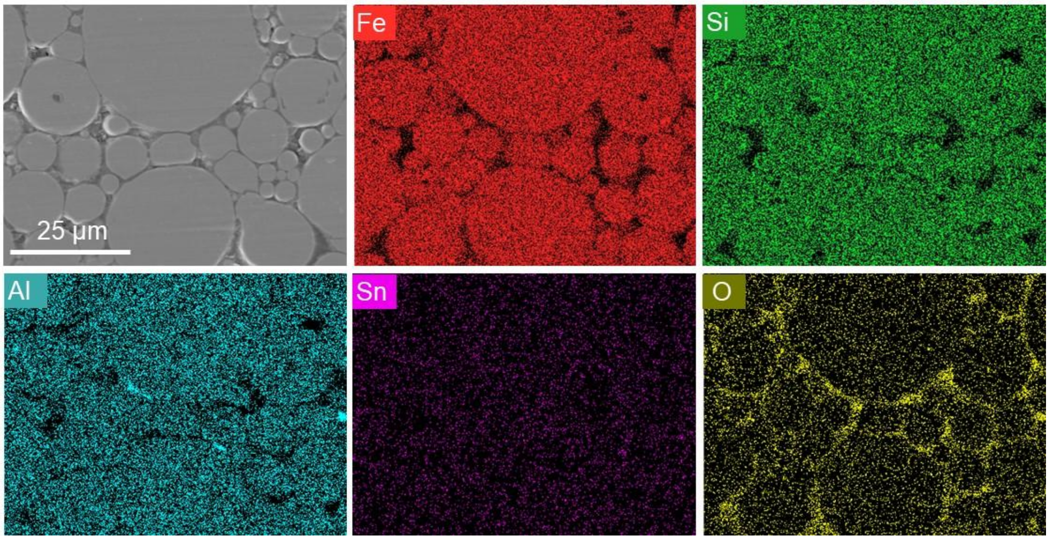

Supplementary Fig. 5. Formation of FeSiAl:Sn and homogeneous oxide layer on FeSiAl particle. Cross sectional SEM and corresponding EDS mapping images of FeSiAl:Sn-0.8 SMC.

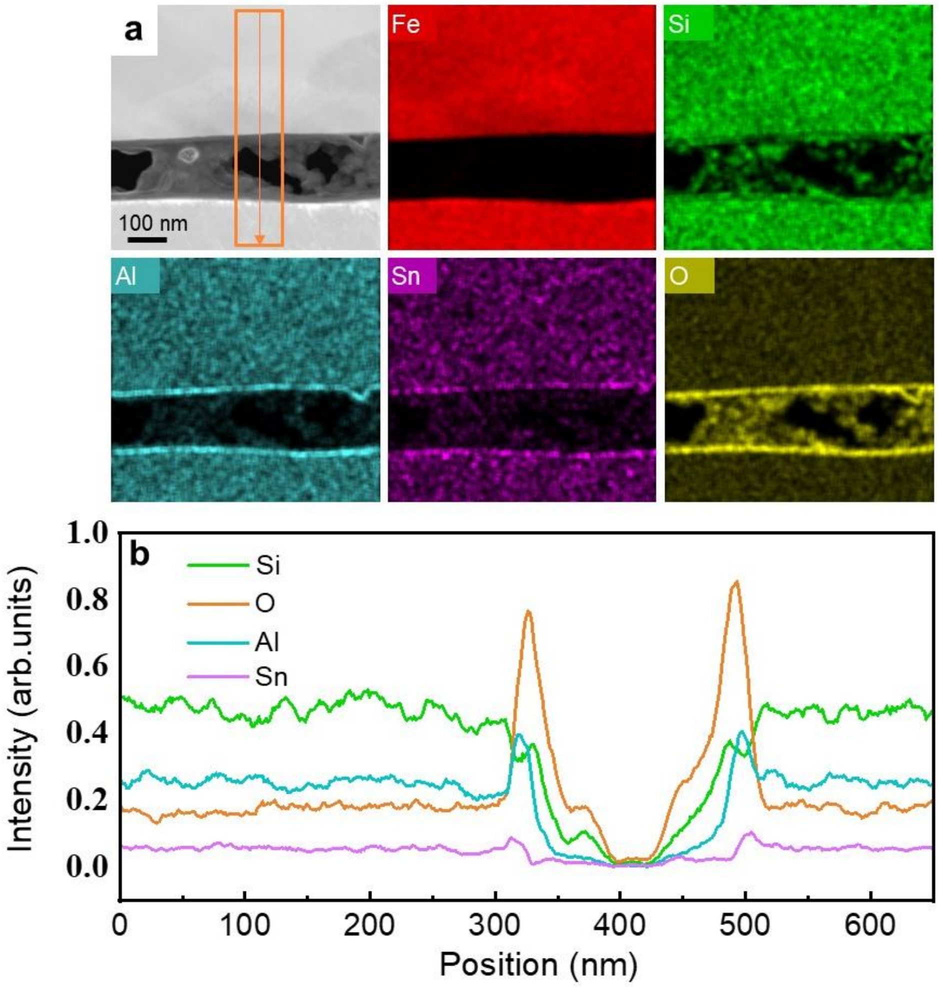

Supplementary Fig. 6. FeSiAl:Sn/Sn-rich/Al_2O_3 structure. a, HAADF image and corresponding EDS mapping of FeSiAl:Sn-0.8 SMC. **b,** The Sn, O, Al and Si intensity profiles along the orange arrow in a. Source data are provided as a Source Data file.

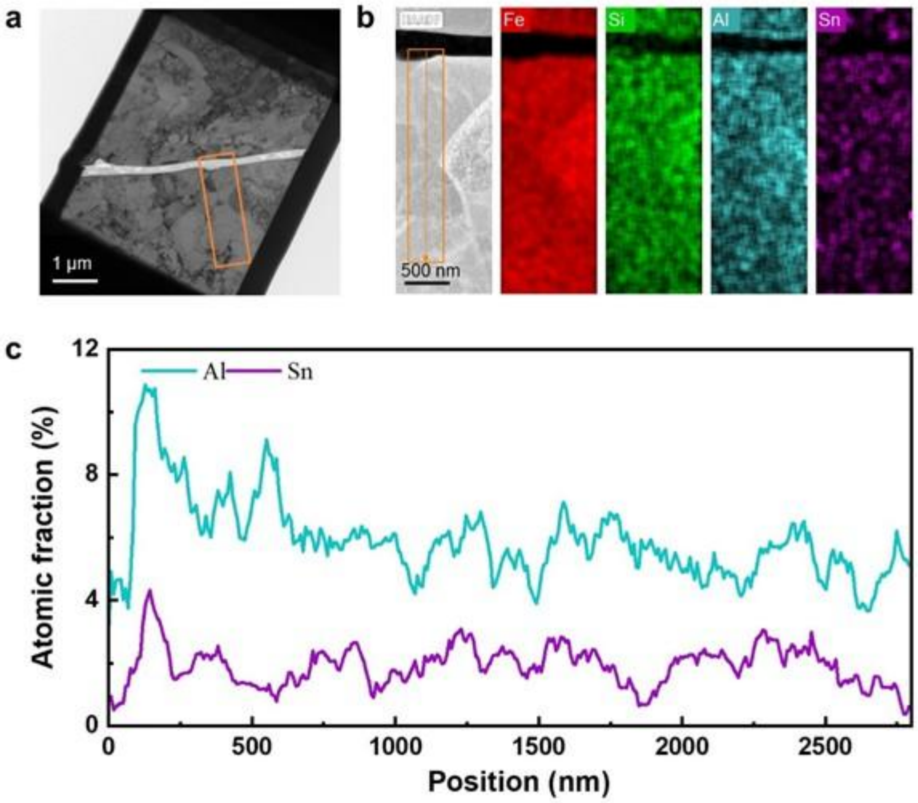

Supplementary Fig. 7. Depth profile of Sn substitution. a, The image of whole FIB sample of FeSiAl:Sn-0.8 SMC. **b,** HAADF and corresponding EDS mapping images of the orange box in a. **c,** The Sn and Al atomic concentration profiles along the cyan arrow in b. Source data are provided as a Source Data file.

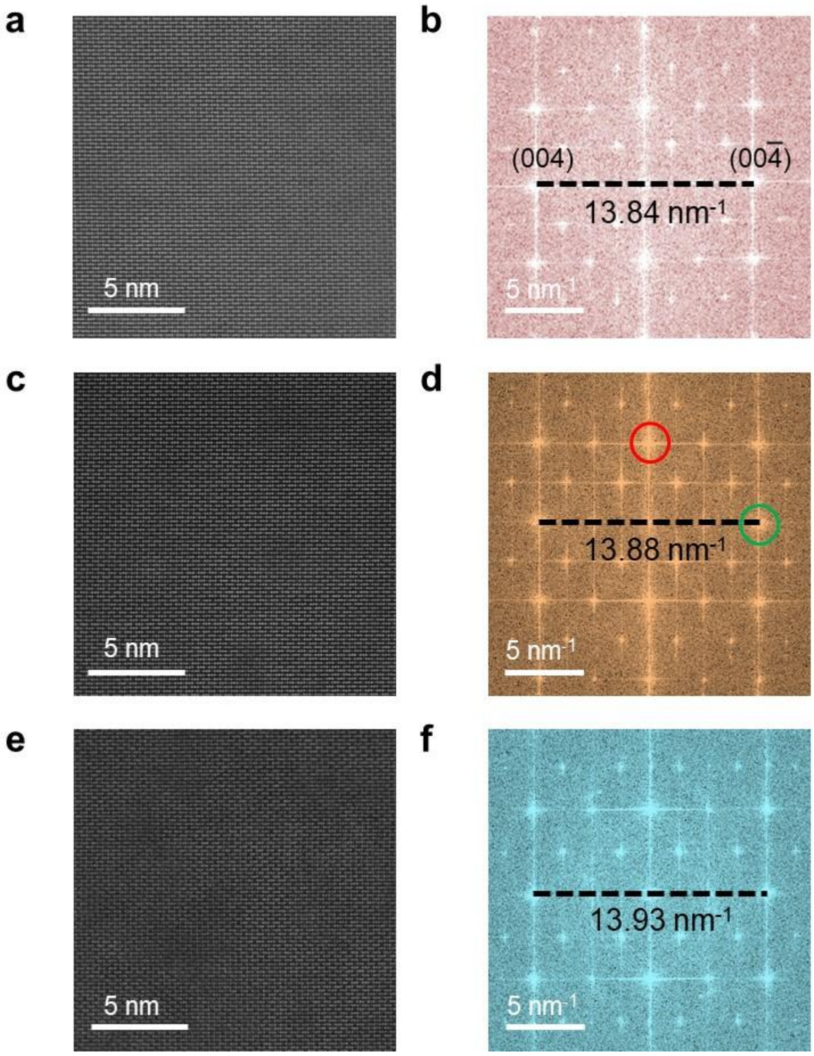

Supplementary Fig. 8. DO3 structured FeSiAl:Sn. a, b, STEM-HAADF image of FeSiAl:Sn matrix of the position I in Fig.3a and the corresponding FFT image. **c, d,** STEM-HAADF image of FeSiAl:Sn matrix of the position II in Fig.3a and the corresponding FFT image. **e, f,** STEM-HAADF image of FeSiAl:Sn matrix of the position III in Fig.3a and the corresponding FFT image. The interplanar spacing of the FeSiAl:Sn matrix exhibits a slight increase from position III to position I due to the gradient substitution of Sn.

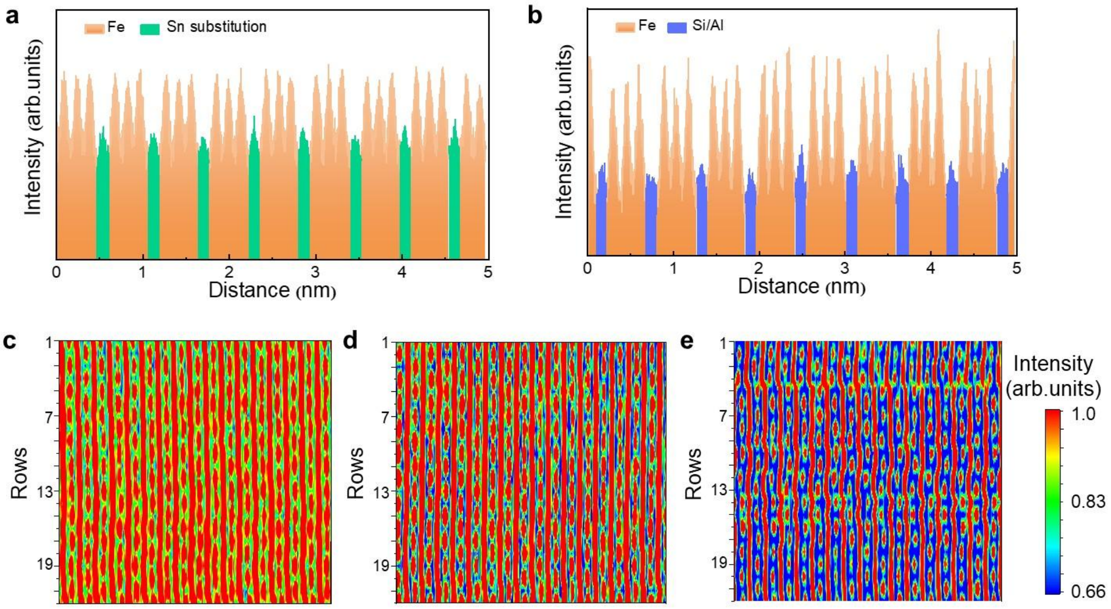

Supplementary Fig. 9. Gradient Sn substitution Al in FeSiAl matrix. a, b, Atomic intensity profiles along the cyan arrow in Fig.3d and Fig.3f, respectively. Strong atomic column intensity (green) is observed at line 17 of position I in comparison with that (blue) of position III, indicating the substitution of Sn for Al. **c-e,** Normalized atomic intensity profiles of Fig.3d**,** Fig.3e and Fig.3f, respectively, confirming the gradient distribution of Sn substitution Al along the depth of the matrix. Source data are provided as a Source Data file.

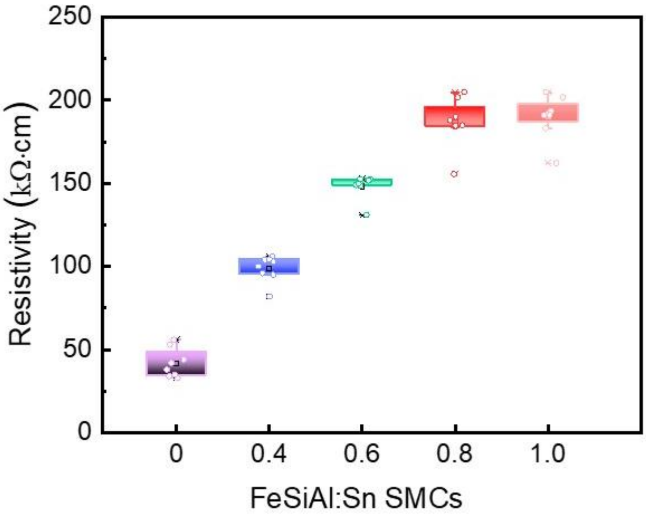

Supplementary Fig. 10. Electrical resistivity of different FeSiAl:Sn SMCs. Source data are provided as a Source Data file.

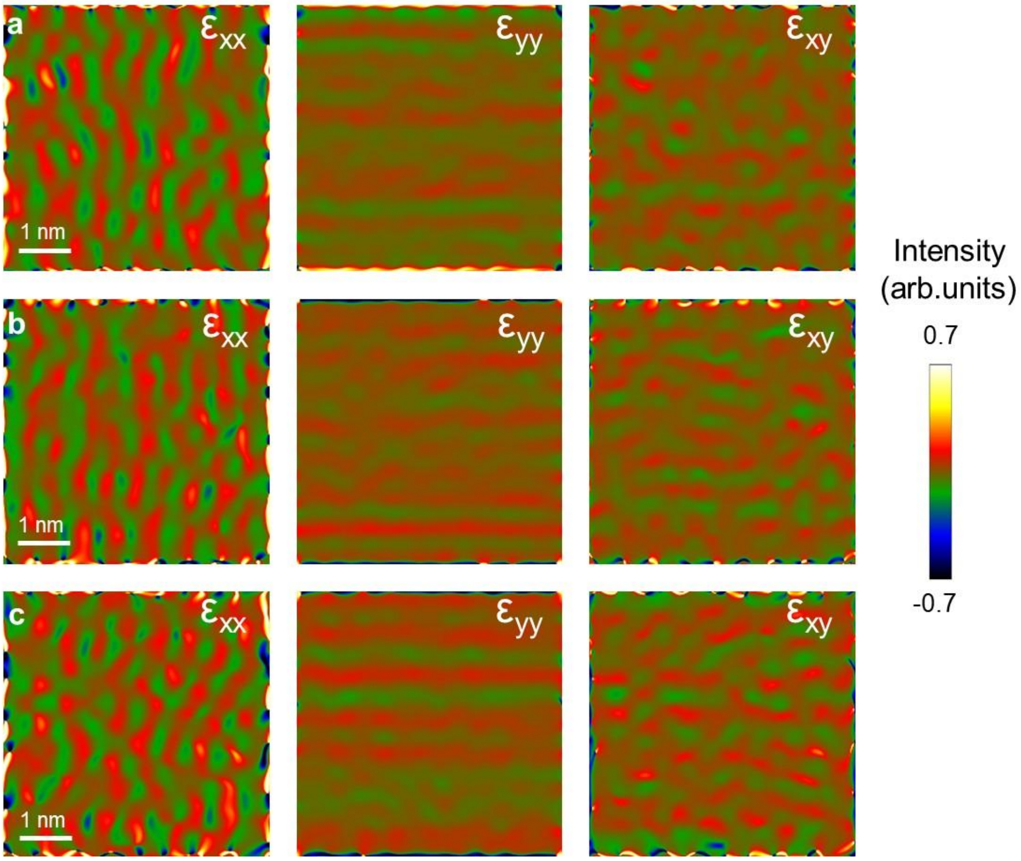

Supplementary Fig. 11. Strain distribution across the FeSiAl:Sn matrix. a, Corresponding GPA strain maps of Fig.3d. **b,** Corresponding GPA strain maps of Fig.3e. **c,** Corresponding GPA strain maps of Fig.3f. The horizontal (x) and vertical (y) directions correspond to the g vectors of the red and green circles in FFT patterns in supplementary Fig.8.

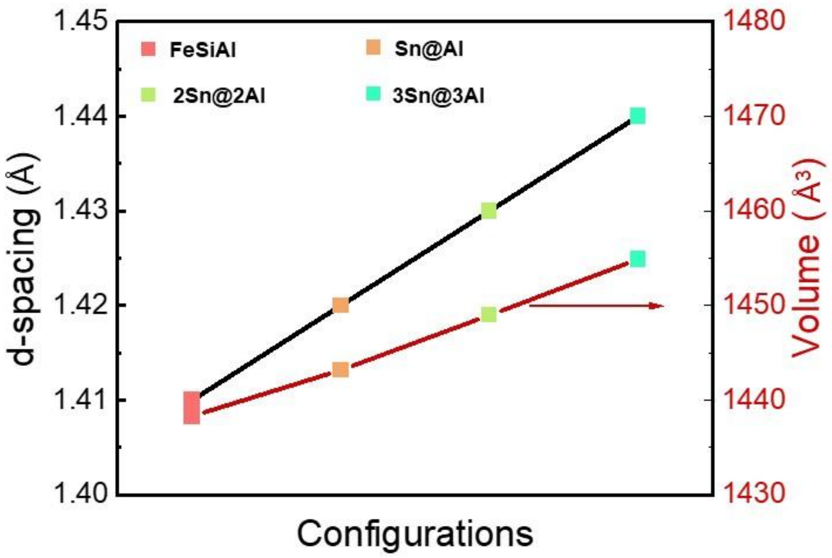

Supplementary Fig. 12. Cell parameters for FeSiAl:Sn structure. The interplanar spacing and cell volume of different models are simulated by first principle. Source data are provided as a Source Data file.

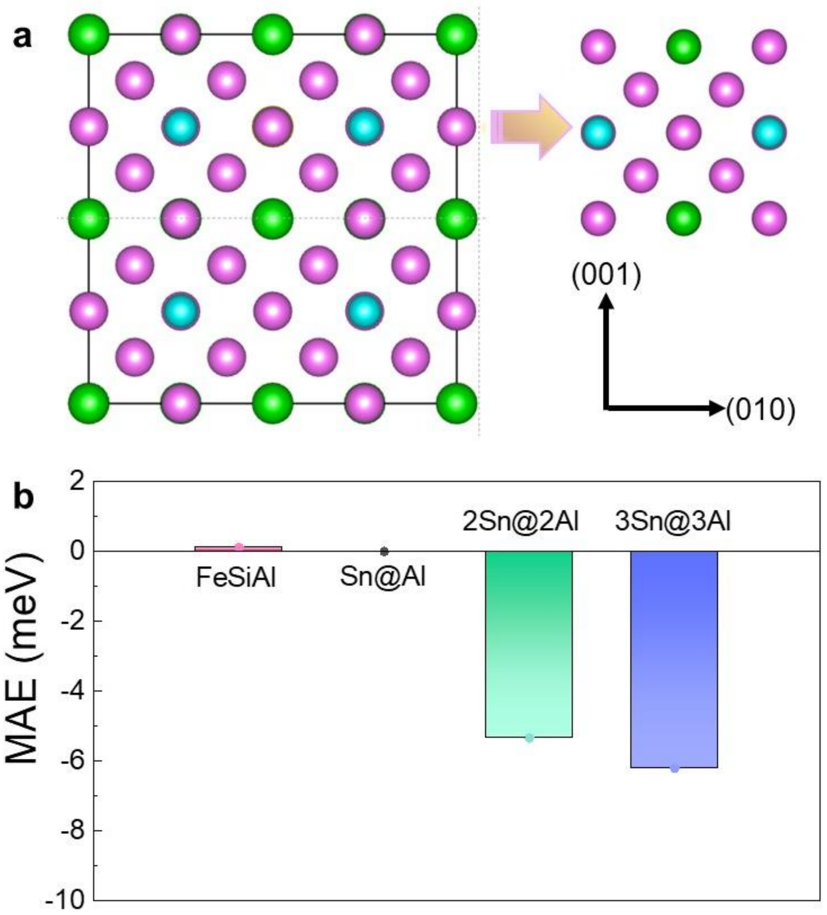

Supplementary Fig. 13. Magneto-crystalline anisotropy energy (MAE) calculation. a, Sketch of the 2×2×2 FeSiAl supercell (left panel) and cubic lattice structure (right panel). **b,** MAE values for different structures with spontaneous magnetization direction along the Z-axis, calculated using density functional theory with a Hubbard U correction (DFT+U) methodology. The MAE is the total energies of the crystal structure with spin orbit coupling, which is defined as $E_{MAE} = E_{[0\,0\,1]} - E_{[1\,0\,0]}$ for the magnetization orientated along the [001] and [100] directions. The absolute value of MAE for Sn@Al is as low as 0.001 meV, indicating a decrease compared with 0.113 meV for FeSiAl. However, a high absolute value of MAE is observed with further increasing the substituted Al content, indicating deterioration of the magneto-crystalline anisotropy. Source data are provided as a Source Data file.

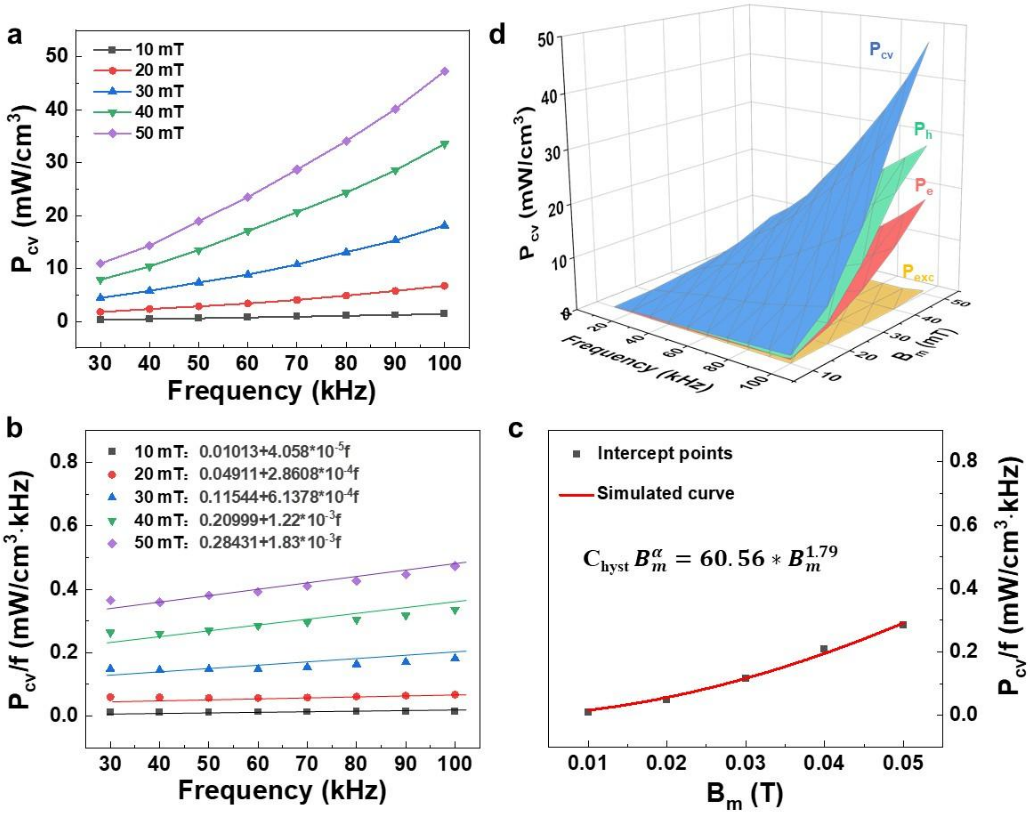

Supplementary Fig. 14. Power loss separation process. (a) Power loss of FeSiAl:Sn-0.8 SMC measured under different B_m. (b) Measured and simulation results for P_{cv}/f-f curve of FeSiAl:Sn-0.8 SMC under different B_m. (c) The intercepts of b on the vertical coordinate with black dots and the simulation curve (red line) for quasi-static hysteresis loss versus B_m. (d) The bulk loss surfaces of power loss, hysteresis loss, eddy current loss and excess loss of FeSiAl:Sn-0.8 SMC, respectively. Source data are provided as a Source Data file.

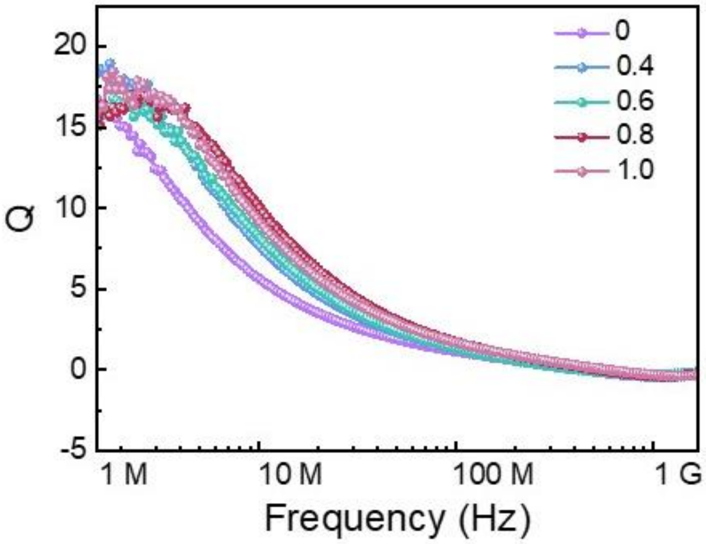

Supplementary Fig. 15. Magnetic performance. Quality factor (Q) of different FeSiAl:Sn SMCs as a function of frequency. Source data are provided as a Source Data file.

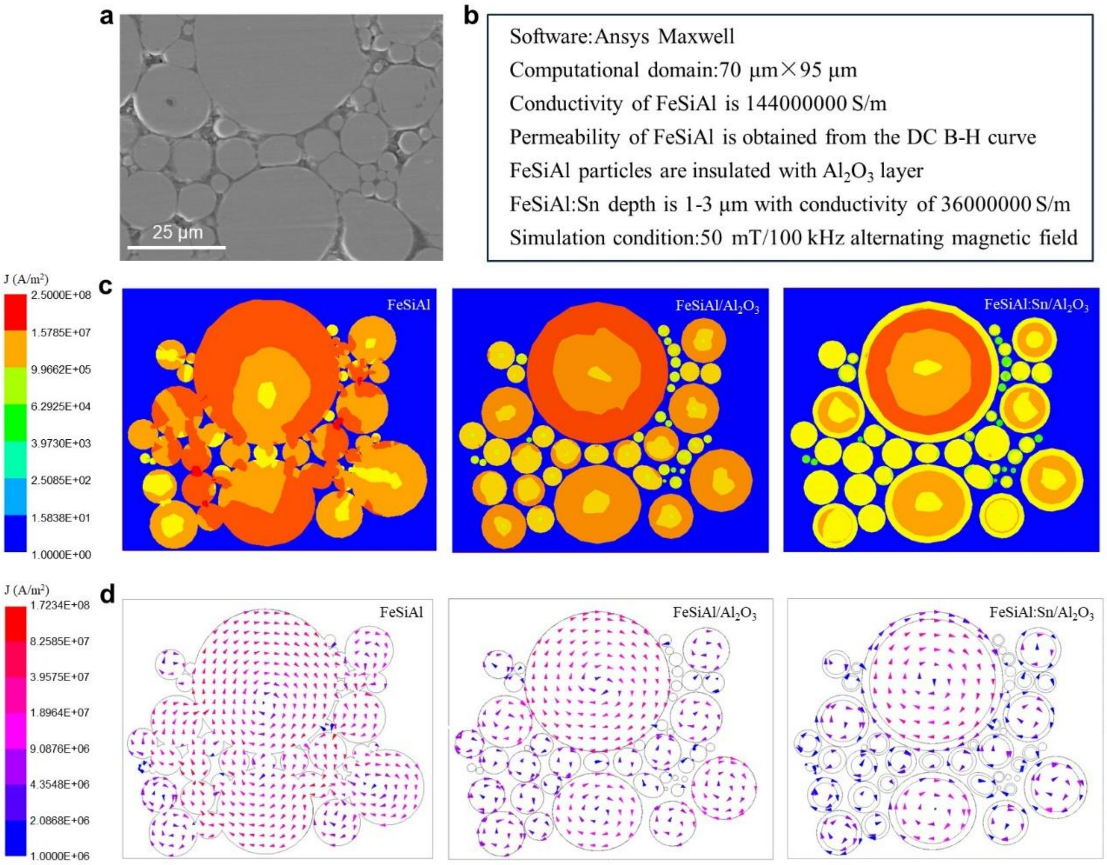

Supplementary Fig. 16. Current density images simulated by Ansys Maxwell. a, Cross sectional SEM image of FeSiAl:Sn-0.8 SMC. **b,** The detailed simulation parameters. **c,** The corresponding simulated current density cloud maps of different SMCs. **d,** The corresponding simulated current density vector maps of different SMCs. The decreased current density is observed for FeSiAl:Sn/Al_2O_3 in comparison with that of FeSiAl and FeSiAl/Al_2O_3 samples.

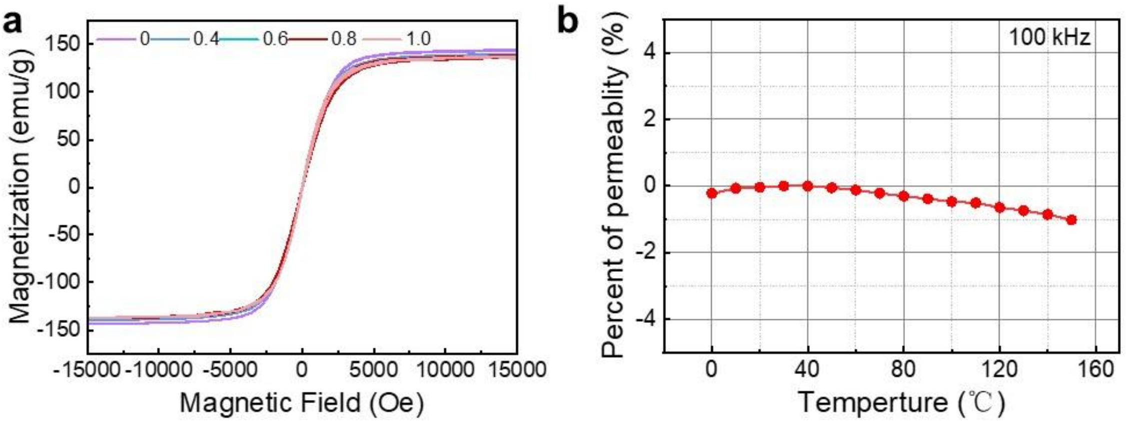

Supplementary Fig. 17. Magnetic performance. a, Hysteresis loops of different FeSiAl:Sn particles after annealing. **b,** Effective permeability as a function of temperature for FeSiAl:Sn-0.8 SMC. Source data are provided as a Source Data file.

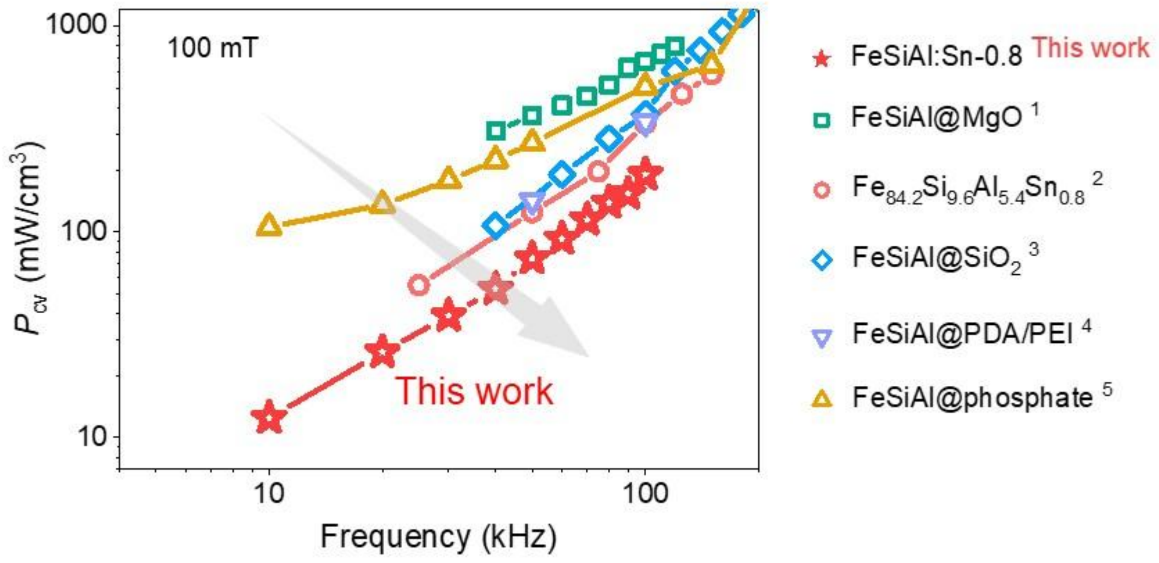

Supplementary Fig. 18. Comparison of power loss of our FeSiAl:Sn-0.8 SMC with other counterparts measured at 100 mT [1-5]. Source data are provided as a Source Data file.

Table S1. Loss separation results of different FeSiAl:Sn SMCs under 50 mT/100 kHz.

SMCs	P_h	P_e	P_h+P_e	P_{cv}
FeSiAl:Sn-0.0	46.8	30.6	77.4	77.63
FeSiAl:Sn-0.4	32.36	24.8	57.16	57.18
FeSiAl:Sn-0.6	32.08	24.6	56.68	56.7
FeSiAl:Sn-0.8	28.4	18.3	46.7	47.3
FeSiAl:Sn-1.0	38.04	18.3	56.34	57.35

Table S2. Comparison of power loss, effective permeability and cut-off frequency (f_r) of FeSiAl:Sn-0.8 SMC with other counterparts.

SMCs	μ_e	P_{cv} (mW/cm^3) 100 mT/ 50 kHz	100 mT/ 100 kHz	50 mT/ 100 kHz	50 mT/ 1 MHz	M_s (emμ/g)	f_r (MHz)
FeSiAl@MgO [1]	37	367	674				
FeSiAl@SiO_2 [3]	42.2		372			118.57	10
FeSiAl@PDA/PEI [4]	53.2	141.7	341	63.8		107.5	
FeSiAl@phosphate [5]	60	270					
$Fe_{84.2}Si_{9.6}Al_{5.4}Sn_{0.8}$ [6]	50.4	124.4		66.3		119.2	
FeSiAl@MoO_3 [7]	29.5			128.8		124	
FeSiAl@H_3PO_4@Na_2SiO_3 [8]	50.9			133.9			
FeSiAl@H_3PO_4 [9]	54.2			142		122.9	
FeSiAl@hBN [10]	40.7		398	100			≥ 30
FeSiAlNi@$ZnFe_2O_4$ [11]	49.6			506.9		145	
FeSiAl@Epoxy resin [12]	36			512		120.8	
FeSiAl@Co_3O_4 [13]	56					105.5	
**FeSiAl:Sn-0.8 [This work]**	**60.2**	**74**	**190**	**47**	**1344**	**136**	**250.7**

Supplementary References:

1. Shi XY, Chen XY, Wan K, Zhang BW, Duan PT, Zhang H, *et al*. Enhanced magnetic and mechanical properties of gas atomized Fe-Si-Al soft magnetic composites through adhesive insulation. *J. Magn. Magn. Mater.*, **534**, 168040 (2021).
2. Liu ZH, Dong YQ, Liu XC, Lu H, Yue Wu, Zhang RH, *et al*. Optimizing soft magnetic properties by reducing internal defects and residual stress of $Fe_{85-x}Si_{9.6}Al_{5.4}Sn_x$ soft magnetic composites. *J. Mater. Res. Technol.*, **18,** 3872-3883 (2022).
3. Li WC. Cai HW, Kang Y, Ying Y, Yu J, Zheng JW, Qiao L, Jiang Y, Che SL. High permeability and low loss bioinspired soft magnetic composites with nacre-like structure for high frequency applications. *Acta Mater.*, **167**, 267-274 (2019).
4. Li XH, Yao HY, Wan YH, Shen FY, Sun YP, Liu XG. The Balance Between Low Core Loss, High Permeability, and Large DC Bias Performance in FeSiAl Cores Covered by Polydopamine/Polyethyleneimine. *J Electro Mater*., **53**, 3128-3142 (2024).
5. Liu HJ, Su HL, Geng WB, Sun ZG. Song TT, Tong XC, Zou Z Q, Wu YC, Du YW. Effect of particle size distribution on the magnetic properties of Fe-Si-Al powder core. *J. Supercond. Nov. Magn.,* **29**, 463-468 (2016).
6. Li G, Cui Y, Zhang N, Wang X, Xie JL. The precipitation in annealing and its effect on permittivity of Fe-Si-Al powders. *Physica B*, **481**, 1-7 (2016).
7. Li Z, Li ZZ, Liu XY, Shi SY, Li HX, Liu XG. Ultra-low core loss FeSiAl-based soft magnetic composites with ultra-thin MoO_3 composite insulating layer. *Ceram. Int.*, **48**, 29705-29714 (2022).
8. Li Z, Li ZZ, Yang H, Li HX, Liu XG. Soft Magnetic properties of gas atomized FeSiAl microparticles with a triple phosphoric acid sodium silicate silicone resin insulation treatment. *J. Electron Mater.*, **51**, 2142-2155 (2022).
9. Li HX, Bai GH, Zhao MY, Yu S, Lu ZW, Yang H, Cheng ML, Zhang ZH, Liu XL, Chen WC, Li Z, Liu XG, Zhang EP, Zhang XF. FeSiAl soft magnetic composite with double Al_2O_3 insulation layers for simultaneous high mechanical and magnetic properties. *J. Mater. Sci. Technol.*, **206**, 307-316 (2025).
10. Ni JL, Duan F, Feng SJ, Hu F, Kan XC, Liu XS. High performance of FeSiAl/hBN soft magnetic composites. *J. Alloy. Compd.,* **897**, 163191 (2022).

11. Li SG, Zhang MH, Zhan ZZ, Liu RT, Xiong X. Study on novel Fe-based core-shell structured soft magnetic composites with remarkable magnetic enhancement by in-situ coating nano-$ZnFe_2O_4$ layer. *J. Magn. Magn. Mater.*, **500**, 166321 (2020).

12. Zhu ZQ, Liu JQ, Zhao H, Pang J. Study of the Soft Magnetic Properties of FeSiAl Magnetic Powder Cores by Compounding with Different Content of Epoxy Resin, *Materials,* **16**, 1270 (2023).

13. Luo Z, Wu W, Jin Q, Yang Z, Li G, Li Y, Fan X, Structural evolution mechanism and magnetic properties of soft magnetic composites transformed from FeSiAl/Co_3O_4 composites, *J. Alloys Compd.,* **1004**, 175819 (2024).
